# Supplementary material for: The burden of cardiovascular disease attributable to dietary risk factors in Australia between 1990 and 2019
Source: PLoS One. 2024 Jan 17;19(1):e0295231. doi: 10.1371/journal.pone.0295231 (PMC10793933; doi:10.1371/journal.pone.0295231)
Supplement: S1 File — (DOCX) [file pone.0295231.s001.docx]

**Supplementary appendix**

# **Selection of dietary risks**

We only included dietary risk factors for which we found convincing or probable evidence on their relationship for Cardiovascular diseases (CVDs). We summarized epidemiologic evidence supporting the causal relationship for the risk-outcome pairs included in our analysis.

# **Estimating the dietary intake (data sources)**

We systematically searched Pubmed to identify studies providing nationally or subnationally representative estimates of consumption of dietary factors. Additionally, we searched the Global Health Data Exchange (GHDx) database for individual-level data from nutrition surveys or household budget surveys providing dietary data. We included all nationally or subnationally representative studies providing data on mean intake of each dietary factor by age and sex. We only included studies reporting data collected between 1 January 1990 and 31 December 2019. Studies were excluded if using non-random samples (e.g., case-control studies or convenience samples); conducted among specific subpopulations (e.g., pregnant women, racial or ethnic minorities, immigrants, or individuals with specific diseases); having sample sizes of less than 20 per 5year age-sex group; providing inadequate information on any of the inclusion criteria. We excluded non-English articles. For seven food groups (fruits, vegetables, legumes, nuts and seeds, red meat, processed meat, SSBs), we used sales data to capture recent trends in the intake. For five food groups (fruits, vegetables, legumes, nuts and seeds, and red meat), we used data from United Nations Food and Agriculture Organization (FAO) food balance sheets (FBS). Additionally, we estimated the national availability of nutrients (i.e., fiber, omega-3 fatty acids, polyunsaturated fatty acids availability, and saturated fatty acids) using data from FAO’s Supply Utilization Accounts (SUA) and the United States Department of Agriculture’s National Nutrition Database for Standard Reference. For whole grains, we used FBS and SUAs data to estimate the availability of refined grains and total grains at the country level and then calculated the availability of whole grains by difference.

We used dietary data from multiple sources and each type of dietary data was affected by specific types of biases. To adjust for biases of each dietary assessment method and to make our dietary data more comparable, we considered 24-hour diet recall as the gold standard method for assessing the mean intake at the population level and adjusted dietary data from other sources accordingly in a subset of countries with data from both methods of assessment. For sodium, 24-hour urinary sodium weas condidered as the gold standard and we converted dietary sodium to urinary equivalent using a multiplier estimated from the surveys reporting both dietary and urinary sodium. Given that some of our data sources (i.e., availability, sales, and household data) were only proving data for all-age groups and both sexes, we first spliut these data into standard from the gold-standard source (i.e., 24-hour diet recall) by age, sex, country, and year and performed a linear regression analysis to estimate the consumption fata from data on availability, sales, and household availability by age, and sex using the following equation:

*Dietary Intake = Availability + age + sex*

# **Relative risk**

We obtained the relative risk of each disease endpoint per serving of the dietary components from recent dose-response meta-analyses of prospective observational studies. Considering the wellestablished age trend of the relative risks of metabolic risk factors for cardiovascular disease, we conducted a literature review to identify the most important metabolic mediators for each dietary factor and used the age trend of the relative risk of that mediator(s) and the disease endpoint to estimate the age-specific relative risk for each dietary factors. We took the following steps to estimate the age-specific relative risk for each dietary factor:

- For each dietary factor, we conducted a systematic review of literature and identified the metabolic risks that could potentially mediate the effect of diet on the disease endpoint.
- We obtained the age-specific relative risk for each mediator and disease endpoint from a meta-analysis of pooled cohort studies.
- For each dietary risk and disease endpoint, we estimated the median age at event from the meta-analysis used to estimate the relative risks of the diet-disease pair.
- We assigned the relative risk reported in the meta-analysis to age group that included median age at event.
- We estimated the percent change in the relative risks between age at event and each group for all relevant metabolic mediators and took the average of them.
- We applied the average percent change in the relative risk of metabolic mediators to the relative risk of the dietary factor at age at event and estimated the age-specific relative risk of the dietary factor.

**Theoretical minimum risk exposure level**

To estimate the TMREL for each dietary factor, we first calculated the level of intake associated with the lowest risk of mortality from each disease endpoint based on the studies included in the meta-analyses of the dietary relative risks. Then, we calculated the TMREL as the weighted average of these numbers using the global number of deaths from each of outcome as the weight. To reflect the uncertainty of TMREL, we have assumed a uniform uncertainty distribution of 20% above and below the mean. For sodium, the evidence supporting the selection of the TMREL is uncertain. Therefore, we included in the uncertainty estimation sampling a uniform distribution of different TMRELs. The manuscript Table provides the range and distribution of the uncertainty in the TMREL.

**Population attributable fraction**

For each dietary risk factor, the attributable burden was estimated by comparing observed health outcomes to those that would have been observed if the past exposure level had been sustained at an optimal level, here referred to as theoretical minimum risk exposure level (TMREL). The main inputs to this analysis included the exposure level (where *l* is the lowest level of intake and *u* is highest level of intake) for each risk factor (p); the effect size of the risk factor on each disease endpoint (RR); the risk factor level associated with the lowest risk (TMREL); and the total number of deaths from each disease endpoint. Using the first three inputs, we estimated the population attributable fraction (PAF) for each risk-disease pair by age (a), sex (s), country (c), and year (t). Then, we used disease-specific (o) PAFs and mortality to calculate the total number of attributable deaths across all relevant outcomes (*w*).

To account for potential overlaps between the effect of foods and nutrients that are associated with the same outcome (e.g., milk and calcium with colorectal cancer), we developed a mediation matrix and only accounted for the effect of either the food item or the nutrient.

Uncertainty of PAF estimates was calculated from 1,000 draws resulting from PAF calculations using the 1,000 draws of exposure estimates, 1,000 draws of relative risk estimates, and 1,000 draws of the theoretical minimum risk exposure level. The 1,000 draws of PAF estimates were multiplied by 1,000 draws of deaths and DALYs to produce 1,000 draws of attributable burden. All components of the PAF calculation analysis were assumed to be independent of each other.

# **GBD modelling methodology and uncertainty**

The DisMod-MR tool evaluated and pooled all available data, adjusted data for systematic bias associated with methods that varied from the reference, and produced estimates by world regions with UIs by using Bayesian statistical methods. In cross-validation tests, the log rates specification worked as well or better than the negative binomial specification. The sequence of estimation occurs at five levels: global, super-region, region, country and, where applicable, subnational location. The super-region priors are generated at the global level with mixed-effects, nonlinear regression by using all available data; the super-region fit, in turn, informs the region fit, and so on down the cascade. The wrapper gives analysts the choice to branch the cascade in terms of time and sex at different levels depending on data density. The default used in most models is to branch by sex after the global fit but to retain all years of data until the lowest level in the cascade is reached. The computational engine is limited to three levels of random effects; we differentiate estimates at the super-region, region and country level. The coefficients for country covariates are re-estimated at each level of the cascade. For a given location, country coefficients are calculated by using both data and prior information available for that location. In the absence of data, the coefficient of its parent location is used to utilise the predictive power of our covariates in data-sparse situations. To determine the robustness of the models, we included the option again to have random effects on cause-specific mortality rates (CSMR) and excess mortality rate (EMR). Based on simulation testing we found that coverage improved and errors reduced when passing down priors with a wider setting of minimum coefficient of variation (which determines the uncertainty around priors and hence how ‘informative’ the priors are) than had generally been used in past GBD iterations. We settled on a default value of 0.8.

We carried out simulation testing using DisMod-MR 2.1 based on an internally consistent set of 15,601 data points for prevalence, incidence, excess mortality, CSMR, and remission. We aimed to test what level of minimum CV would create the best fit based on the following three performance statistics:

(1) Coverage, ie, the proportion of data point mean values that fall between the 2.5th and 97.5^th^ percentile of the draws of the fit values; (2) Root mean square error: the square root of the mean of the squares of the difference between data point mean values and the mean fit value; and (3) Bias: the difference between the mean fit value and the data point mean value.

We created different datasets culling the initial complete set with values at every age, sex, and location to more realistic data sparsity scenarios for analysis.

A first strategy was to randomly reduce the dataset to 10%, 5%, 2.5%, 1%, and 0.5% of the original data points. Initial results indicated little variation between the data samples culled to 10%, 5%, 2.5%, and 1%. The 0.5% culled dataset was an exception with markedly worse performance statistics, particularly with regard to bias and RMSE. We conducted further studies using the datasets culled to 10%, 5%, and 0.5%.

For some diseases with a range of sequelae differentiated by severity, such as COPD or diabetes mellitus, DisMod-MR 2.1 was used to meta-analyse the data on overall prevalence with separate DisMod-MR 2.1 models of the proportions of cases with different severity levels or sequelae.

We extensively examine the potential sources of measurement bias and, if we find evidence for a systematic bias between an alternative case definition and our reference, we make data adjustments before running our models as a way of adjusting for confounding. In addition, we run Bayesian meta-regression models to make those adjustments between data sources with differently measured input data based on matching pairs of data within a study or between data sources for the same age, sex, location and year category. By using statistical adjustments for key covariates and harmonisation of between-study heterogeneity by formally using a reference definition, our modelled data provide the most accurate estimates of dietary data in Australia addressing limitations of previous studies.

**Definition of health metrices for cardiovascular diseases**

| Health metrices | Definition |
| --- | --- |
| Years lived with a disability (YLDs) | Years of life lived with any short-term or long term health loss |
| Years of life lost (YLLs) | Years of life lost due to premature mortality |
| Disability adjusted years (DALYs) | The sum of years lost due to premature death (YLLs) and years lived with disability (YLDs). DALYs are also defined as years of healthy life lost |
| Death (mortality) | Refers to the state of being mortal |

**List of Group of twenty countries (G20)**

| Group of twenty countries (G20) | Argentina, Australia, Brazil, Canada, China, France, Germany, India, Indonesia, Italy, Japan, Republic of Korea, Mexico, Russia, Saudi Arabia, South Africa, Turkey, United Kingdom, United States, and European Union |
| --- | --- |

**Supplementary Table 1**: List of cardiovascular disease (CVD) investigated.

| **Number** | **Cardiovascular diseases** |
| --- | --- |
| B.2 | CVDs (inclusive of all conditions) |
| B.2.1 | Rheumatic heart disease |
| B.2.2 | Ischemic heart disease |
| B.2.3 | Stroke |
| B.2.3.1 | Ischemic stroke |
| B.2.3.2 | Intracerebral haemorrhage |
| B.2.3.3 | Subarachnoid haemorrhage |
| B.2.4 | Hypertensive heart disease |
| B.2.5 | Non-rheumatic valvular heart disease |
| B.2.5.1 | Non-rheumatic calcific aortic valve disease |
| B.2.5.2 | Non-rheumatic degenerative mitral valve disease |
| B.5.3 | Other non-rheumatic valve diseases |
| B.2.6 | Cardiomyopathy and myocarditis |
| B.2.6.1 | Myocarditis |
| B.2.6.2 | Alcoholic cardiomyopathy |
| B.2.6.3 | Other cardiomyopathy |
| B.2.8 | Atrial fibrillation and flutter |
| B.2.9 | Aortic aneurysm |
| B.2.10 | Peripheral artery |
| B.2.11 | Endocarditis |
| B.2.12 | Other cardiovascular and circulatory diseases |

**Supplementary Table 2:** Dietary risk factors, exposure definition and optimal level (TMREL) in the Australian adult population.

| Number | Dietary risk factor | Exposure definition | Theoretical minimum risk exposure level per adult |
| --- | --- | --- | --- |
| 1 | Diet low in fruits | Average daily intake of fruits (fresh, frozen, cooked, canned, or dried fruits, excluding, fruits juices and salted or pickled fruits) | Consumption of less than 3 servings (11 ounces total) of fruits per day |
| 2 | Diet low in vegetables | Average daily intake of vegetables (fresh, frozen, cooked, canned, or dried vegetables, excluding legumes and salted or pickled vegetables, juices, nuts, and seeds, and starchy vegetables and such as potato or corn | Consumption of less than 4 servings (14 ounces) of vegetables per day |
| 3 | Diet low in legumes | Average daily intake of legumes (fresh, frozen, cooked, canned, or dried legumes) | Consumption of less than 50 grams of legumes per day |
| 4 | Diet low in wholegrains | Average daily intake of wholegrains (bran, germ, and endosperm in their natural proportion) from breakfast cereals, bread, rice, pasta. Biscuits, muffins, tortillas, pancakes, and other sources | Consumption of less than 2.5 servings (4 ounces) per day of wholegrains |
| 5 | Diet low in nuts and seeds | Average daily intake of nuts and seeds | Consumption of less than 4 servings (4 ounces) of nuts and seeds per week |
| 6 | Diet high in red meat | Mean daily consumption of red meat (beef, pork, lamb, and goat, but excluded poultry, fish, eggs, and all processed meats) | Consumption of more than 1 serving (4 ounces) per week |
| 7 | Diet high in processed meat | Average daily intake of meat preserved by smoking, curing, salting, or addition of chemical preservatives | Consumption of any processed meat per day |
| 8 | Diet high in sugar-sweetened beverages | Average daily intake of beverages with > 50 kcal per 226.8 g serving, including carbonated beverages, sodas, energy drinks, fruits, but excluding 100% fruit and vegetable juices | Consumption of any beverage with ≥50 calories per day |
| 9 | Diet low in fibre | Average daily intake of fibre from all sources including fruits, vegetables, grains, legumes,  and pulses. | Consumption of less than 30 grams of fibre per day |
| 10 | Diet low in seafood omega-3 fatty acids | Average daily intake of eicosatetraenoic acid and docosahexaenoic acid | Less than 250 milligrams per day |
| 11 | Diet low in polyunsaturated fatty acids | Average daily intake of omega-6 fatty acids from all sources, mainly liquid vegetables oils, including soybean oil, corn oil, and safflower oil | Less than 12% of total calories from polyunsaturated fatty acids per day |
| 12 | Diet high in trans fatty acids | Average daily intake of trans fat from all sources, mainly partially hydrogenated vegetable oils and ruminant products | Consumption of more than 0.5% of total calories from trans fatty acids per day |
| 13 | Diet high in sodium | 24 h urinary sodium measured in g/day | 24 h urinary sodium of more than 1,000 milligrams per day |

**Supplementary Table 3**: Changes (95% uncertainty interval [UI]) in rates per 100,000 cardiovascular disease deaths, years lived with disability (YLDs), years of life lost (YLLs), and disability-adjusted life years (DALYs) attributable to overall and specific dietary risk factors in Australia, by sex, between 1990 and 2019.

| **Dietary risk factors** | **Sex** | **Change (95% UI) in rates of deaths** | **Change (95% UI) in rates of YLDs** | **Change (95% UI) in rates of YLLs 2019** | **Change (95% UI) in rates of DALYs** |
| --- | --- | --- | --- | --- | --- |
| All risk factors combined | Men | -68.4% (-70.5, -66.4) | -37.1% (-42.4, -30.9) | -70.5% (-72.3, -68.7) | -69.4% (-71.2, -67.4) |
|  | Women | -68.3% (-71.4, -65.7) | -33.1% (-38.9, -27.0) | -72.5% (-74.7, -70.4) | -69.9% (-72.0, -67.5) |
| Diet low in fruit | Men | -69.6% (-74.4, -65.0) | -38.9% (-46.8, -31.4) | -71.3% (-74.6, -67.5) | -69.9% (-73.4, -65.8) |
|  | Women | -69.2% (-75.1, -63.3) | -31.2% (-41.2, -22.0) | -72.2% (-76.2, -67.0) | -68.6% (-73.3, -62.1) |
| Diet low in vegetables | Men | -71.3% (-76.4, -67.4) | -45.5% (-54.9, -38.0) | -73.8% (-78.8, -70.1) | -72.9% (-77.8, -69.0) |
|  | Women | -71.1% (-76.7, -66.3) | -42.4% (-55.8, -31.9) | -75.5% (-80.2, -71.1) | -73.3% (-78.5, -68.1) |
| Diet low in legumes | Men | -69.5% (-72.9, -67.7) | -42.4% (-51.6, -35.7) | -71.7% (-76.2, -69.7) | -71.0% (-75.7, -69.0) |
|  | Women | -70.4% (-74.3, -67.9) | -49.5% (-58.7, -43.0) | -75.0% (-78.8, -72.7) | -74.1% (-78.1, -71.9) |
| Diet low in wholegrains | Men | -66.9% (-69.3, -64.2) | -34.6% (-39.7, -28.2) | -68.7% (-70.8, -66.2) | -67.7% (-69.8, -65.3) |
|  | Women | -67.2% (-70.6, -63.1) | -31.9% (-40.7, -22.6) | -71.7% (-74.0, -68.8) | -69.6% (-72.2, -65.9) |
| Diet low in nuts and seeds | Men | -73.9% (-81.4, -60.0) | -56.7% (-69.2, -35.2) | -78.3% (-84.0, - 68.2) | -77.8% (-83.6, -67.5) |
|  | Women | -72.8% (-84.2, -51.3) | -62.3% (-75.9, -38.4) | -79.2% (-86.6, -65.7) | -78.6% (-86.2, -64.7) |
| Diet high in red meat | Men | -67.4% (-71.7, -61.8) | -34.4% (-40.7, -27.2) | -69.2% (-72.2, -65.4) | -67.6% (-70.7, -63.5) |
|  | Women | -66.4% (-72.4, -58.9) | -25.4% (-31.9, -17.17) | -70.1% (-73.7, -65.3) | -65.6% (-69.8, -59.3) |
| Diet high in processed meat | Men | -62.6%, (-70.2, -38.9) | -34.4% (-40.7, -27.2) | 63.1% (-70.5, -39.8) | -69.8% (-69.8, -38.6) |
|  | Women | -66.2% (-74.9, 44.0) | -25.4% (-31.9, -17.2) | -70.0% (-75.6, -53.4) | -68.9% (-74.9, -51.8) |
| Diet high in SSB | Men | -66.3% (-75.9, -51.5) | -36.3% (-53.0, -13.5) | -67.2% (-74.8, -55.7) | -66.3% (-74.3, -54.7) |
|  | Women | -67.9% (-79.8, -45.1) | -44.2% (-61.7, -19.1) | -71.5% (-80.0, -56.7) | -70.6% (-79.3, -55.6) |
| Diet low in fibre | Men | -78.6% (-82.9, -74.1) | -50.1% (-58.1, -42.1) | -71.8% (-76.7, -65.5) | -76.6% (-80.1, -72.6) |
|  | Women | -73.5% (-79.1, -67.3) | -39.8% (-51.2, -30.8) | -75.2% (-79.6, -69.8) | -73.4% (-80.1, -72.6) |
| Diet low in omega 3 fatty acids | Men | -69.4% (-75.2, -62.6) | -43.1% (-54.3, -27.7) | -71.8% (-76.7, -65.5) | -71.1% (-76.1, -64.7) |
|  | Women | -70.7% (-76.8, -63.4,) | -50.4% (-60.1, -38.6) | -75.2% (-79.6, -69.8) | -74.3% (-78.9, -68.8) |
| Diet low in PUFA | Men | -69.9% (-73.3, -67.8) | -42.3% (-50.8, -36.1) | -71.7% (-74.9, -69.8) | -71.0% (-74.3, -69.1) |
|  | Women | -71.0% (-74.7, -68.4) | -49.2% (-56.8, -42.5) | -75.1% (-77.9, -73.1) | -74.2% (-77.2, -72.2) |
| Diet high in trans fatty acids | Men | -72.0% (-74.8, -69.1) | -46.6% (-53.3, -39.5) | -73.8% (-76.5, -71.2) | -73.2% (-75.9, -70.6) |
|  | Women | -72.9% (-76.5, -69.6) | -53.0% (-59.9, -45.4) | -76.8% (-79.5, -74.4) | -76.0% (-78.7, -73.5) |
| Diet high in sodium | Men | -64.6% (-78.5, -41.2) | -24.8% (-59.9, -20.1) | -66.00% (-81.6, -43.5) | -63.6% (-80.2, -39.4) |
|  | Women | -61.3% (-81.6, -24.5) | -18.3% (-58.4, -34.1) | -65.4% (-83.2, -40.0) | -60.3% (-80.9, -33.2) |

***Note*** a negative sign (-) indicates decrease in the rates.

**Supplementary Table 4**: Changes (95% uncertainty interval [UI]) in rates per 100,000 of overall and major cardiovascular disease deaths, years lived with disability (YLDs), years of life lost (YLLs), and disability-adjusted life years (DALYs) attributable to dietary risk factors in Australia, by sex, between 1990 and 2019.

| **Cardiovascular disease** | **Sex** | **Change (95% UI) in rates of deaths** | **Change (95% UI) in rates of YLDs** | **Change (95% UI) in rates of YLLs 2019** | **Change (95% UI) in rates of DALYs** |
| --- | --- | --- | --- | --- | --- |
| All Cardiovascular diseases | Men | -68.4% (-70.5, -66.4) | -37.1% (-42.4, -30.9) | -70.5% (-72.3, -68.7) | -69.4% (-71.2, -67.4) |
|  | Women | -68.3% (-71.4, -65.7) | -33.1% (-38.9, -27.0) | -72.5% (-74.7, -70.4) | -69.9% (-72.0, -67.5) |
| Ischemic heart disease | Men | -69.3% (-71.3, -67.4) | -41.2% (-46.8, -34.8) | -71.3% (-73.1, -69.7) | -70.7% (-72.3, -69.0) |
|  | Women | -70.2% (-73.1, -67.9) | -48.3% (-54.4, -41.9) | -74.7% (-76.7, -72.8) | -73.7% (-75.7, -71.8) |
| Stroke | Men | -63.2% (-67.4, -58.8) | -34.5% (-41.4, -26.5) | -65.5% (-69.1, -61.6) | -61.8% (-65.3, -58.1) |
|  | Women | -59.7% (-64.3, -55.1) | -23.3% (-30.2, -15.7) | -63.5% (-67.1, -59.6) | -56.0% (-59.7, -52.1) |
| Ischemic stroke | Men | -69.0% (-73.3, -64.3) | -37.3% (-45.6, -28.4) | -73.6% (-76.9, -69.9) | -67.3% (-72.7, -63.5) |
|  | Women | -62.3% (-67.7, -57.1) | -24.2% (-32.6, -15.3) | -68.2% (-72.1, -64.1) | -56.2% (-61.0, -51.5) |
| Intracerebral haemorrhage | Men | -55.9% (-62.1, -49.5) | -31.0% (-43.0, -17.0) | -60.4% (-65.9, -54.6) | -59.2% (-64.7, -53.5) |
|  | Women | -57.6% (-63.3, -52.3) | -24.8% (-37.6, -11.2) | -62.8% (-67.2, -58.1) | -59.9% (-64.3, -55.3) |

***Note*** a negative sign (-) indicates decrease in the rates.

**Supplementary Table 5**: Changes (95% uncertainty interval [UI]) in rates per 100,000 cardiovascular disease deaths, years lived with disability (YLDs), years of life lost (YLLs), and disability-adjusted life years (DALYs) attributable to dietary risk factors in Australia, by age groups and sex, between 1990 and 2019.

| **Age** | **Sex** | **Change (95% UI) in rates of deaths** | **Change (95% UI) in rates of YLDs** | **Change (95% UI) in rates of YLLs 2019** | **Change (95% UI) in rates of DALYs** |
| --- | --- | --- | --- | --- | --- |
| 25–29 years | Men | -56.0% (-66.8, -44.2) | -13.2% (-36.6, 19.1) | -56.0% (-66.9, -40.3) | -51.7% (-62.4 -36.6) |
|  | Women | -68.6% (-77.2, -56.7) | -7.8% (-41.3, 48.2) | -68.7% (-77.2, -56.7) | -50.9% (-63.6, -35.5) |
| 30–34 years | Men | -48.9% (-60.2, -34.7) | -15.9% (-47.1, 41.1) | -49.0% (-60.2, -34.8) | -46.6% (-57.7, -32.9) |
|  | Women | -56.3% (-67.3, -42.6) | -9.1% (-19.5, -0.2) | -56.4% (-67.4, -42.7) | -44.8% (-54.7, -33.1) |
| 35–39 years | Men | -52.6% (-61.4, -41.8) | -18.5% (-46.5, 20.7) | -52.7% (-61.4, -41.8) | -51.0% (-59.5, -40.6) |
|  | Women | -49.0% (-59.8, -36.5) | -10.8% (-38.1, 27.1) | -49.0% (-59.8, -36.5) | -41.2% (-51.8, -29.3) |
| 40–44 years | Men | -56.2% (-63.0, -48.3) | -25.3% (-47.1, 4.2) | -56.2% (-63.0, -48.3) | -54.9% (-61.8, -47.1) |
|  | Women | -48.4% (-58.0, -37.3) | -19.6% (-42.7, 11.6) | -48.3% (-58.0, -37.2) | -43.6% (-52.8, -32.4) |
| 45–49 years | Men | -61.7% (-67.4, -55.5) | -27.4% (-45.5, -4.1) | -61.6% (-67.3, -55.5) | -60.4% (-66.1, -54.0) |
|  | Women | -58.2% (-66.6, -48.9) | -17.6% (-38.7, 10.7) | -58.1% (-66.5, -48.8) | -53.3% (-61.6, -43.8) |
| 50–54 years | Men | -66.5% (-70.9, 61.8) | -31.3% (-46.4, -11.4) | -66.5% (-70.8, -61.7) | -65.4% (-69.8, -60.6) |
|  | Women | -69.1% (-74.2, 63.0) | -21.9% (-39.1, 1.6) | -69.1% (-74.2, -62.9) | -64.9% (-70.2, -58.6) |
| 55–59 years | Men | -72.8% (-76.3, 68.9) | -34.0% (-47.6, -16.0) | -72.8% (-76.3, -68.8) | -71.7% (-75.2, -67.7) |
|  | Women | -75.5% (-79.4, -70.9) | -24.6% (-39.6, -5.8) | -75.5% (-79.4, 70.9) | -71.8% (-76.0, -66.6) |
| 60–64 years | Men | -76.6% (-79.5, -73.0) | -40.4% (-52.2, -26.1) | -76.6% (-79.6, -73.0) | -75.6% (-78.6, -72.0) |
|  | Women | -81.1% (-83.7, 77.8) | -36.4% (-48.2, -21.5) | -81.1% (-83.7, -77.8) | -78.4% (-81.3, 74.8) |
| 65–69 years | Men | -78.6% (-81.1, -75.9) | -41.3% (-50.9, -29.4) | -78.6% (-81.2, 75.9) | -77.4% (-80.1, -74.4) |
|  | Women | -83.0% (-85.6, -80.0) | -39.6% (-48.4, -27.7) | -83.0% (-85.6, -80.0) | -80.3% (-83.0, -77.1) |
| 70–74 years | Men | -78.2% (-80.6, -75.5) | -41.5% (-51.1, -28.9) | -78.2% (-80.6, -75.5) | -76.9% (-79.4, -74.0) |
|  | Women | -82.6% (-84.9, -79.9) | -42.2% (-50.9, -30.9) | -82.6% (-84.9, -79.9) | -80.3% (-82.8, -77.4) |
| 75–79 years | Men | -76.4% (-79.2, -73.5) | -42.5% (-52.1, -30.5) | -76.6% (-79.3, -73.7) | -75.3% (-78.1, -72.2) |
|  | Women | -79.1% (-82.1, -75.9) | -44.8% (-52.9, -34.3) | -79.2% (-83.1, -75.9) | -77.4% (-80.4, -74.0) |
| 80–84 years | Men | -70.1% (-73.7, -66.3) | -44.6% (-53.0, -33.6) | -70.4% (-74.0, -66.6) | -69.4% (-72.8, -65.7) |
|  | Women | -72.4% (-76.2, -68.4) | -45.1% (-53.5, -35.9) | -72.6% (-76.4, -68.6) | -71.2% (-74.9, -67.3) |
| 85–89 years | Men | -55.8% (-62.2, -48.6) | -41.0% (-52.2, 25.7) | -56.2% (-62.5, -49.0) | -55.55% (-61.7, -48.3) |
|  | Women | -60.4% (-66.0, -53.8) | -43.9% (-55.0, -30.8) | -60.6% (-66.2, -54.1) | -59.8% (-65.4, -53.2) |
| 90–94 years | Men | -49.4% (-56.6, -41.9) | -37.9% (-50.2, -22.5) | -49.7% (-56.8, -42.2) | -49.2% (-56.3, -41.8) |
|  | Women | -48.5% (-55.35, -41.3) | -38.7% (-51.2, 24.5) | -48.8% (-55.6, -41.6) | -48.3% (-55.1, -41.1) |
| ≥95 years | Men | -42.5% (-50.3, -33.6) | -34.9% (-49.0, -19.0) | -44.2% (-51.7, -35.5) | -43.9% (-51.4, -35.4) |
|  | Women | -37.0% (-44.8, -27.8) | -36.0% (-48.9, -21.1) | -37.9% (-45.7, -28.9) | -37.9% (-45.6, -29.0) |

***Note*** a negative sign (-) indicates decrease in the rates.
