# Supplementary material for: The burden of cardiovascular disease attributable to dietary risk factors in Australia between 1990 and 2019
Source: PLoS One. 2024 Jan 17;19(1):e0295231. doi: 10.1371/journal.pone.0295231 (PMC10793933; doi:10.1371/journal.pone.0295231)
Supplement: Attachment — Submitted filename: Response to reviewers.docx [file pone.0295231.s002.docx]

**The burden of cardiovascular disease attributable to dietary risk factors in Australia between 1990 and 2019**

**General comments**

| Please include the following items when submitting your revised manuscript:   - A rebuttal letter that responds to each point raised by the academic editor and reviewer(s). You should upload this letter as a separate file labeled 'Response to Reviewers'. - A marked-up copy of your manuscript that highlights changes made to the original version. You should upload this as a separate file labeled 'Revised Manuscript with Track Changes'. - An unmarked version of your revised paper without tracked changes. You should upload this as a separate file labeled 'Manuscript'. | Response to reviewers has been included.  Marked-up and unmarked versions of the manuscript have been submitted separately. |
| --- | --- |
| 1. Please ensure that your manuscript meets PLOS ONE's style requirements, including those for file naming. The PLOS ONE style templates can be found at  <https://journals.plos.org/plosone/s/file?id=wjVg/PLOSOne_formatting_sample_main_body.pdf> and  <https://journals.plos.org/plosone/s/file?id=ba62/PLOSOne_formatting_sample_title_authors_affiliations.pdf> | Manuscript has been reformatted to fully comply with the PLOS ONE style requirements as per the templates. |
| 2. Thank you for stating the following in the Funding Section of your manuscript:  “The GBD Study is funded by the Bill and Melinda Gates Foundation.”  We note that you have provided funding information that is not currently declared in your Funding Statement. However, funding information should not appear in the Acknowledgments section or other areas of your manuscript. We will only publish funding information present in the Funding Statement section of the online submission form.  Please remove any funding-related text from the manuscript and let us know how you would like to update your Funding Statement. Currently, your Funding Statement reads as follows:  “The authors received no specific funding for this work.”  Please include your amended statements within your cover letter; we will change the online submission form on your behalf. | Funding-related text has been removed from the manuscript as suggested and included in the cover letter. |
| 3. In your Data Availability statement, you have not specified where the minimal data set underlying the results described in your manuscript can be found. PLOS defines a study's minimal data set as the underlying data used to reach the conclusions drawn in the manuscript and any additional data required to replicate the reported study findings in their entirety. All PLOS journals require that the minimal data set be made fully available. For more information about our data policy, please see <http://journals.plos.org/plosone/s/data-availability>.  Upon re-submitting your revised manuscript, please upload your study’s minimal underlying data set as either Supporting Information files or to a stable, public repository and include the relevant URLs, DOIs, or accession numbers within your revised cover letter. For a list of acceptable repositories, please see <http://journals.plos.org/plosone/s/data-availability#loc-recommended-repositories>. Any potentially identifying patient information must be fully anonymized.  Important: If there are ethical or legal restrictions to sharing your data publicly, please explain these restrictions in detail. Please see our guidelines for more information on what we consider unacceptable restrictions to publicly sharing data: <http://journals.plos.org/plosone/s/data-availability#loc-unacceptable-data-access-restrictions>. Note that it is not acceptable for the authors to be the sole named individuals responsible for ensuring data access.  We will update your Data Availability statement to reflect the information you provide in your cover letter. | We have now provided in the cover letter information re access of the minimal data set:  “The datasets used in the current study are publicly available at https://vizhub.healthdata.org/gbd-results/. Data were extracted using the GBD Compare and GBD Results tool. (https://www.healthdata.org/data-tools-practices/interactive-visuals/gbd-compare)” |
| 4. We notice that your supplementary tables are included in the manuscript file. Please remove them and upload them with the file type 'Supporting Information'. Please ensure that each Supporting Information file has a legend listed in the manuscript after the references list. | These have now been uploaded as a separate file as per your instruction. |

**Reviewer 1**

| This interesting paper uses global burden of disease methodology to assess the contribution of poor diet to CVD risk in Australia over the period 1990-2019. Note that it would be helpful in reviewing this work if line numbers were included. The results are valuable to inform health promotion messages. The analysis is explained in detail, but I am not able to adequately assess the detail of the methodology. | Thank you for the kind comments.  Line numbers have now been added.  Details of the methodologies have been previously published and referenced in the manuscripts. See: Vos T, Lim SS, Abbafati C, et al. Global burden of 369 diseases and injuries in 204 countries and territories, 1990–2019: a systematic analysis for the Global Burden of Disease Study 2019. Lancet 2020;396(10258):1204–22. |
| --- | --- |
| “Of the 13 dietary risk factors assessed, diets high in sodium for women and diets high in processed meat for men observed the lowest decrease in percentage change for age-standardised rate for CVD deaths,” This is from page 6 and I wonder whether there is something wrong here with a decrease in percentage change. | No this is correct.  It is an expected finding that CVD mortality decrease will be lower in people that consume high sodium and high processed meat diets. |
| In the second para of the discussion changes in diet in Aus might not be sufficient to account for changes in diet-related CVD burden. Does this make sense in terms of the way the GBD works? | The GBD estimates the burden and trends of diseases and risk factors over time and allows comparisons in change of diseases and risk factors from previous rounds using population level data and robust modellings. While the burden of diet-related CVD in Australia decreased since 1990, the changes in dietary pattern observed during this period (i.e. diet high in sodium for women and processed meat for men) suggests that the changes might not be due to dietary changes alone, rather attributable to other factors such as decrease in smoking, increased physical activity etc. |
| Figure 5 in the Whitnall and Pitts reference shows that intake of sheep and beef meat declined in Aus and poultry intake increased dramatically during 1998-2018 which does not seem consistent with the statement “Over the past three decades, the intake of red meat has increased among the Australian population, 30” attributed to this reference. | Thank you for picking this up. We have reworded this statement (lines 301-305):  “Over the past three decades, the intake of red meat has decreased among the Australian population while that of poultry has dramatically increased.^30^ However, reports from 2011/12 show that the average intake of red meat was 565 grams per week per person aged ≥2 years, which exceeds the Heart Foundation recommendation of 350 grams of unprocessed red meat per week.^31^” |
| Bottom of page 9-page 10, here you talk about targeting “diet low in sodium, wholegrains, legumes, and high in red meat and trans-fatty acids,” why is sodium grouped with things to eat more of rather than things to eat less of? | We have now reworded this statement to (lines 353-356):  “However, our results suggest that it may be beneficial to promote a diet high in wholegrains and legumes, and low in red meat and trans-fatty acids, in addition to lowering sodium intake.” |

**Reviewer 2**

| Overall, this manuscript presents an important investigation into the burden of cardiovascular diseases (CVDs) attributed to dietary risk factors in Australia over a 30-year period. While the study is valuable and well-structured, there are several areas where improvements are needed before it can be considered for publication. The review is constructive and provides specific recommendations for enhancing the manuscript. | Thank you for your kind comments and feedback to improve the manuscript. |
| --- | --- |
| 1. Clarity and Organization: - The manuscript's overall structure is clear, with sections such as the abstract and introduction providing a concise overview. However, the presentation of results and discussion can be improved for better clarity and coherence. It would be beneficial to reorganize and rewrite certain sections to ensure a smoother flow of information. | Thank you for the comment. We have presented the Results in sections for clarity and discussed the main results including comparison with findings from current literature, possible explanations of our findings with limitations and future recommendations. Please let us know if you have specific recommendations regarding the organisation of sections. |
| 2. Data Sources and Limitations: - The manuscript mentions that dietary data were sourced from various surveys and organizations. However, the authors should provide more specific details regarding these sources, including their representativeness, sample sizes, and potential biases. Understanding the quality of the dietary data is critical for the reader's confidence in the results. - The limitations section should be expanded to discuss how the limitations in dietary data may have influenced the findings and the interpretation of the study. Addressing these limitations would enhance the manuscript's transparency. | All data sources specific to Australian dietary data used to generate the GBD 2019 estimates in this manuscript are publicly available online in GBD data sources. The GBD 2019 data completeness, quality, and limitation have been described elsewhere (Ref 1, 2) and the GBD protocol, including the detail methodology is available online. (https://ghdx.healthdata.org/gbd-2019).  Unfortunately, these lists do not contain information about the validity or completeness of the data sources, but more information about these data sources are available in GBD study protocol and GBD capstone papers published in The Lancet.  *References:*  1. GBD 2019 Diseases and Injuries Collaborators. Global burden of 369 diseases and injuries in 204 countries and territories, 1990–2019: a systematic analysis for the Global Burden of Disease Study 2019. Lancet 2020; 396: 1204–22.  2. <https://ghdx.healthdata.org/gbd-2019/data-input-sources>.  We have added the following statement in the limitations (lines 329-331):  “Dietary data collected from surveys and observational studies are prone to self-reporting biases and measurement errors. These inherent limitations may have biased analyses and interpretations presented in this study.” |
| 3. Statistical Methods and Uncertainty: - While the manuscript explains the statistical methods employed, it would be beneficial to elaborate on the assumptions made during modeling and discuss the potential implications of these assumptions on the results. Providing a more comprehensive discussion of uncertainties would enhance the robustness of the findings. | Thank you for the comment. We regret that space limitations preclude describing our methods more comprehensively in the main text. We have included information in the Appendix about the modelling process and uncertainty estimation process:  The GBD modelling software has been previously validated by comparing projected estimates with observed data for 2014-16. Model performance was assessed using mean error and root-mean-squared error. The model performed better than the most commonly used approach, i.e. the Lee-Carter method. We have added details on the DisMod-MR and model evaluation in the revised manuscript (Supplementary file).  The DisMod-MR tool evaluated and pooled all available data, adjusted data for systematic bias associated with methods that varied from the reference, and produced estimates by world regions with UIs by using Bayesian statistical methods. In cross-validation tests, the log rates specification worked as well or better than the negative binomial specification. The sequence of estimation occurs at five levels: global, super-region, region, country and, where applicable, subnational location. The super-region priors are generated at the global level with mixed-effects, nonlinear regression by using all available data; the super-region fit, in turn, informs the region fit, and so on down the cascade. The wrapper gives analysts the choice to branch the cascade in terms of time and sex at different levels depending on data density. The default used in most models is to branch by sex after the global fit but to retain all years of data until the lowest level in the cascade is reached. The computational engine is limited to three levels of random effects; we differentiate estimates at the super-region, region and country level. The coefficients for country covariates are re-estimated at each level of the cascade. For a given location, country coefficients are calculated by using both data and prior information available for that location. In the absence of data, the coefficient of its parent location is used to utilise the predictive power of our covariates in data-sparse situations. To determine the robustness of the models, we included the option again to have random effects on cause-specific mortality rates (CSMR) and excess mortality rate (EMR). Based on simulation testing we found that coverage improved and errors reduced when passing down priors with a wider setting of minimum coefficient of variation (which determines the uncertainty around priors and hence how ‘informative’ the priors are) than had generally been used in past GBD iterations. We settled on a default value of 0.8.  We carried out simulation testing using DisMod-MR 2.1 based on an internally consistent set of 15,601 data points for prevalence, incidence, excess mortality, CSMR, and remission. We aimed to test what level of minimum CV would create the best fit based on the following three performance statistics:  (1) Coverage, ie, the proportion of data point mean values that fall between the 2.5th and 97.5^th^ percentile of the draws of the fit values; (2) Root mean square error: the square root of the mean of the squares of the difference between data point mean values and the mean fit value; and (3) Bias: the difference between the mean fit value and the data point mean value.  We created different datasets culling the initial complete set with values at every age, sex, and location to more realistic data sparsity scenarios for analysis.  A first strategy was to randomly reduce the dataset to 10%, 5%, 2.5%, 1%, and 0.5% of the original data points. Initial results indicated little variation between the data samples culled to 10%, 5%, 2.5%, and 1%. The 0.5% culled dataset was an exception with markedly worse performance statistics, particularly with regard to bias and RMSE. We conducted further studies using the datasets culled to 10%, 5%, and 0.5%.  For some diseases with a range of sequelae differentiated by severity, such as COPD or diabetes mellitus, DisMod-MR 2.1 was used to meta-analyse the data on overall prevalence with separate DisMod-MR 2.1 models of the proportions of cases with different severity levels or sequelae.  We extensively examine the potential sources of measurement bias and, if we find evidence for a systematic bias between an alternative case definition and our reference, we make data adjustments before running our models as a way of adjusting for confounding. In addition, we run Bayesian meta-regression models to make those adjustments between data sources with differently measured input data based on matching pairs of data within a study or between data sources for the same age, sex, location and year category. By using statistical adjustments for key covariates and harmonisation of between-study heterogeneity by formally using a reference definition, our modelled data provide the most accurate estimates of dietary data in Australia addressing limitations of previous studies. |
| 4. Comparison with Other Studies: - The manuscript claims to be the first to investigate trends in CVDs attributable to dietary risk factors in Australia. While this is an important contribution, it would be valuable to contextualize the findings by comparing them with similar studies in other countries or regions. Discussing the consistency or divergence of results would provide a broader perspective. | Thank you for this comment. We have expanded the section in the Discussion of the manuscript where we contextualize the findings by comparing them to countries and regions with similar SES, such as countries of the European Union, the US, and Asian countries of similar SES. (Lines 310-322):  “Compared to countries or regions with similar socio-demographics or socio-economics (i.e. the United States of America and countries of the European Union), Australia was among the nations with the highest rate of CVD burden for high red meat intake over the last three decades.^5^ Our finding is in agreement with previous studies reporting red meat intake being the main contributor for CVD burden, including mortality and DALYs.^32^ In contrast, CVD deaths from high sodium intake in Australia remained low between 1990 and 2019.^5^ Similarly, a study in countries of the European region reported that sodium was the fourth leading risk factor for CVD mortality, behind diets low in whole grains, nuts and seeds, and fruits.^33^ Whereas a study in Asian countries of similar sociodemographics ranked sodium as the third leading risk factor for CVD burden, again behing a diet low in whole grains, as well as diets low in legumes.^34^ In our study, ischemic heart disease was the leading CVD attributable to dietary risk factors during 1990 and 2019, which is also the leading diet-related CVD globally.^35”^ |
| 5. Sex and Age Differences: - The manuscript touches upon differences in CVD burden between sexes and age groups but does not delve deeply into the potential reasons for these disparities. It would be beneficial to include discussions about the socio-cultural, economic, and behavioral factors that might contribute to these differences, providing a more comprehensive analysis. | Thank you for the comment. Although we agree with your comment that socioeconomic and behavioural factors might contribute to these differences, these are not available in the GBD data sets. Age is certainly associated with certain behaviours, e.g. exercise level declines with age but it was beyond the scope of this manuscript to include all effect modifiers. Therefore, we chose to focus on sex and age, to also be consistent with other published GBD papers. |
| 6. Policy Implications: - The manuscript concludes by suggesting the need for dietary policies but lacks specific recommendations or discussions on the feasibility and potential challenges of implementing such policies. A more detailed exploration of the implications of the findings for public health policy and potential interventions is warranted. | We have added the following sentence in the discussion (Lines 359-361):  “Future studies are warranted to assess the effectiveness of such dietary changes on CVD outcomes, as well as their feasibility and any potential challenges or barriers to their implementation.” |
| 7. Accessibility and Reproducibility: - The manuscript should include a clear reference to the data sources and analytical methods used to ensure transparency and reproducibility. This is crucial for researchers who may wish to replicate or build upon the study. | Thank you for the comment. All data sources, codes and methodology used in this study are publicly available online. The GBD 2019 data completeness, quality, and limitation have been described elsewhere (Ref 16) and the GBD protocol, including the detail methodology is available online. The GBD online data visualisation tools can be used to explore additional results from our study, and these data can be downloaded using GBD results query tool.  The datasets used in the current study are publicly available at https://vizhub.healthdata.org/gbd-results/. Data were extracted using the GBD Compare and GBD Results tool. (https://www.healthdata.org/data-tools-practices/interactive-visuals/gbd-compare) |
| Overall, this manuscript makes a valuable contribution to understanding the burden of CVDs attributable to dietary risk factors in Australia. However, to enhance its quality and readiness for publication, significant revisions are necessary. The authors should consider reorganizing and rewriting certain sections, providing more specific details about data sources and limitations, conducting sensitivity analyses, comparing findings with other studies, exploring reasons for sex and age differences, and discussing policy implications in greater depth. Addressing these recommendations will strengthen the manuscript and its potential impact on public health. | We thank you for the review and important feedback. We have revised the manuscript in terms of reorganising the sections including methodology, rewritten certain sections, provided more details about data sources and limitations, compared findings with similar studies, explored reasons for age and sex differences and discussed policy implications as suggested.  We agree that sensitivity analyses could have added novelty to the manuscript, but this is beyond our capacity and not the focus of our work. The intent of our paper was to highlight the burden of CVD attributable to CVD in Australia. We have noted this in the Discussion section and that it should be included in future research. (lines 343-345):  “Finally, apart from stratifying for age and gender we have not conducted other sensitivity analyses, future research could include these.” |
